# What Components of Working Memory Are Impaired in Children with Reading and/or Mathematics Difficulties?

**DOI:** 10.3390/children10101719

**Published:** 2023-10-23

**Authors:** Rui Chen, George K. Georgiou, Peng Peng, Yuanyuan Li, Beilei Li, Jiali Wang, Sha Tao

**Affiliations:** 1 State Key Laboratory of Cognitive Neuroscience and Learning, Beijing Normal University, Beijing 100875, China; 202031061015@mail.bnu.edu.cn (R.C.); 202331061064@mail.bnu.edu.cn (Y.L.); 202121061064@mail.bnu.edu.cn (J.W.); 2 IDG/McGovern Institute for Brain Research, Beijing Normal University, Beijing 100875, China; 3 Department of Educational Psychology, University of Alberta, Edmonton, AB T6G 2G5, Canada; 4 Department of Special Education, College of Education, The University of Texas at Austin, Austin, TX 78712, USA; pengpeng@austin.utexas.edu; 5 Faculty of Education, Beijing Normal University, Beijing 100875, China; beileili@bnu.edu.cn

**Keywords:** working memory, learning disabilities, comorbidity, reading, mathematics

## Abstract

Both reading difficulties (RD) and mathematics difficulties (MD) are common neurodevelopmental disorders. The co-occurrence of RD and MD, known as comorbid RDMD, is estimated to range between 21% and 45% of children with learning disabilities. Deficits in working memory have been reported in both RD and MD groups, as well as among comorbid RDMD. However, previous comorbidity studies have only examined the role of some components of working memory, and they do not strictly match their groups on relevant reading and mathematics tasks. Thus, the purpose of this study is to examine the nature of working memory deficits in comorbid RDMD after matching groups based on reading and mathematics tasks. We assessed four groups of children (RD [*n* = 21, *M*_age_ = 10.96 years], MD [*n* = 24, *M*_age_ = 11.04 years], comorbid RDMD [*n* = 26, *M*_age_ = 10.90 years], and chronological-age controls [*n* = 27, *M*_age_ = 10.96 years]) on measures of the phonological loop (word span and digit span forward tasks), central executive (complex word and digit span), and updating tasks (word and digit 2-back). The results of ANCOVA (covarying for gender and non-verbal IQ) showed first that the RD and RDMD groups performed significantly worse than the MD and control groups in both measures of the phonological loop. For the central executive and updating tasks, we found an effect based on stimulus type. For word-related tasks, the RD and comorbid RDMD groups performed worse than the MD and control groups, and for number-related tasks, the MD and comorbid RDMD groups performed worse than the RD and control groups. Taken together, our findings provide support for the correlated liability model of comorbidity, which indicates that working memory deficits experienced by the RDMD group are an additive combination of deficits observed in the RD and MD groups, suggesting that working memory tasks used to examine underlying deficits in reading and/or mathematics difficulties may dictate whether or not significant group differences are found.

## 1. Introduction

Reading difficulties (RD) and mathematics difficulties (MD) are neurodevelopmental disorders commonly observed among school-age children [1,2]. The co-occurrence of RD and MD, known as comorbid RDMD, is estimated to range between 21% and 45% of children with learning disabilities [3,4]. These disabilities have long-lasting negative effects on educational attainment and professional success [5]. A critical role in the process of acquiring reading and mathematical skills has been assigned to working memory[6,7], which is the capacity to store information for a short period of time and manipulate or process it [8,9]. In two recent meta-analytic studies, Peng and colleagues [10,11] reported an average correlation of *r* = 0.29 between working memory and reading and *r* = 0.35 between working memory and mathematics. Similarly, in their meta-analysis, Peng and Fuchs [12] found that children with learning disabilities experience difficulties in their working memory.

According to Baddeley [8,9], working memory (WM) comprises a supervisory system, known as the central executive, as well as specialized temporary memory systems, such as the phonologically based memory store (known as the phonological loop) and the visuospatial memory store (known as the visuospatial sketchpad) [13]. In addition, some researchers have argued that updating, which involves the dynamic processing of contents in the working memory by replacing outdated information with new and relevant information, should also be considered a part of working memory [14,15]. The phonological loop is usually assessed with simple span tasks (e.g., the WISC-R digit span forward task), central executive with complex span tasks (e.g., the Daneman-Carpenter listening or reading span task), and updating with N-back tasks. Because the results concerning the visuospatial sketchpad are clear (i.e., they show that it is specifically impaired in children with mathematics difficulties [16,17,18]), we will not discuss them further.

To date, even though several studies have examined the role of different components of working memory in children with RD or MD [19,20,21,22], only a few studies have examined these components in comorbid RDMD groups and have some important limitations. First, previous studies lack proper matching in relevant reading and mathematics tasks among different groups, potentially influencing their results [23,24,25,26,27]. For example, as seen in Moll et al. [26], the comorbid RDMD groups performed worse than the MD group in mathematics and worse than the RD group in reading. This means that the lower performance of the RDMD group in working memory tasks might be due to the fact that the RDMD group already has worse performance in reading and mathematics than single deficit groups. Second, previous studies with comorbid RDMD groups did not include tasks with both word and numerical stimuli [26,27,28]. Arguably, if children in the RD group have difficulties in reading, they may experience more severe deficits in reading-related memory tasks (e.g., word span task), and MD children may experience more severe deficits in working memory tasks with numerical stimuli (e.g., digit span task). A recent meta-analysis conducted by Peng et al. [12] found that children with MD and comorbid RDMD showed a greater number of severe deficits in numerical working memory tasks than RD children. Finally, previous comorbidity studies on working memory did not measure all components of working memory, especially updating [23,25,26,27,29].

Two models have been proposed in the literature to explain the working memory deficits observed in comorbid RDMD: the correlated liability model [25,27] and the three independent disorders model [30]. The correlated liability model posits that the comorbidity of RDMD may arise from the association of potential risk factors for RD and MD, whereas RD and MD are independent difficulties [31,32]. Consequently, children with comorbid RDMD show an additive combination of RD and MD deficits in their working memory. Researchers have tested whether these effects are additive or not by examining the significant interaction between reading difficulties and mathematics difficulties. If the interaction is not significant, this indicates that the effects are additive [28,33]. This model has received some support from studies involving the phonological loop and central executive [25,27,28,33,34]. For example, Peters et al. [34] found that the effects of reading difficulties were significant for digit span, whereas the effects of mathematics difficulties and their interaction were not, indicating that the difficulties faced by the comorbid RDMD group are mainly additive from deficits of the single difficulties groups. By contrast, three independent disorders model views of comorbid RDMD found distinctive difficulties with risk factors that were, at least in part, dissimilar from those contributing to RD and MD [31,32]. Therefore, the comorbid RDMD group may exhibit over-additive deficiencies in working memory (as indicated by significant RD × MD interactions). So far, only a limited number of studies have provided evidence in favor of the three independent disorders model. For instance, Weerdt et al. [25] observed significant interactions between the RD and the MD group in a complex word span task (listening recall), while Landerl et al. [33] showed significant interactions using a digit span forward task.

In this study, we aim to examine working memory deficits in children with single and comorbid difficulties in China in an attempt to shed light on what comorbidity model best characterizes working memory deficits in the RDMD group. In doing so, we carefully matched the RDMD group to other disability groups based on reading and mathematics tasks, and we also used both word- and number-related stimuli in working memory tasks. We hypothesized that the comorbid group would exhibit deficits in all domains and components of working memory and that the effects would be additive.

An important feature of this study is that it was conducted in China. To the best of our knowledge, only two studies have examined the role of working memory in RD, MD, and RDMD groups in China and have produced mixed findings [27,35]. Working with a group of 12- to 15-year-old children, Deng et al. [35] showed that only the RDMD group exhibited deficits in the phonological loop (word span and digit span forward tasks), central executive (digit span backward task) and updating tasks (object n-back task), while no significant differences were observed between the RD and MD groups and the control group. By contrast, working with a group of first-graders, Wong et al. [27] found that none of the groups with disabilities showed deficits in central executive tasks (syllable span backward task). Thus, more research is needed on the role of different working memory components in groups with reading and mathematics difficulties in China.

## 2. Materials and Methods

### 2.1. Participants

We selected our participants in a stepwise fashion. First, with the permission of the school principals and head teachers of each participating class, we sent letters of information to the families of all 812 children attending Grade 5 in five public elementary schools in Beijing, China. Among the 812 children, 805 children received parental consent and were subsequently tested in their classroom on a reading (Chinese Character Recognition Measure and Assessment Scale for Primary School Children; [36]) and a math (Math Computation from the Wide Range Achievement Test-4; [37]) task. The seven children who did not receive parental consent were asked to read a book so that they would not feel left out. Using the total sample of 805 children as the reference group, children with RD were selected if they scored below the 20th percentile on character recognition and above the 35th percentile on the math computation test. Children with MD were selected if they scored below the 20th percentile on the math computation test and above the 35th percentile on the character recognition test. Children with comorbid RDMD had to score below the 20th percentile on both the character recognition and math computation tests. Finally, the control group was selected based on performance above the 35th percentile in both reading and mathematics. The non-verbal IQ score of all participants had to be above the 25th percentile in Raven’s Matrices. After applying these selection criteria, our sample consisted of 98 children (21 children with RD, 24 children with MD, 26 children with comorbid RDMD, and 27 children with no deficits in either reading or mathematics). Sample characteristics are shown in Table 1.

A series of ANOVAs with the Group (control, RD, MD, and comorbid RDMD) as a fixed factor showed that all groups were comparable in age, *F* (3, 94) = 0.547, *p* = 0.654, η_p_^2^ = 0.017, non-verbal IQ, *F* (3, 94) = 0.567, *p* = 0.638, η_p_^2^ = 0.018, and mother*’*s education level, χ^2^(3) = 0.391, *p* = 0.942 (see Table 1). For gender, the control group had more girls than each of the MD and RDMD groups, χ^2^(3) = 13.823, *p* < 0.01. Gender and non-verbal IQ were controlled in the subsequent analyses. As expected, children with reading difficulties (RD, comorbid RDMD) performed worse on the reading tests compared to children without reading difficulties (MD, Control) (*p*s < 0.001). No statistically significant differences were observed between the MD and control groups, as well as between the RD and comorbid RDMD groups on the reading tests. In turn, children with mathematics difficulties (MD, comorbid RDMD) performed worse on mathematics tests compared to children without mathematics difficulties (RD, Control) (*p*s < 0.001). Also, no statistically significant differences were observed between the RD and control groups, as well as between the MD and comorbid RDMD groups for the mathematics tests.

### 2.2. Tasks and Procedure

The tests of non-verbal IQ, math computation, and Chinese character recognition were administered to the whole classroom. Individual tests of Chinese character identification, math problem solving, and working memory tasks were administered by trained research assistants in a quiet room at school in three 60-min sessions. All working memory tasks were administered in a random order for each child. Ethical approval was granted by the Institutional Review Boards at Beijing Normal University in accordance with the declaration of Helsinki. Informed consent was obtained from parents, and approval was received from the school principals and teachers. All study procedures were reviewed and approved.

#### 2.2.1. Non-Verbal IQ

We used the Chinese version of Raven’s Progressive Matrices [38] to measure the non-verbal IQ. This test consisted of 60 items of increasing difficulty. Children were shown an array of shapes and geometric designs that were interrelated within a visual matrix and had a missing piece. Children were subsequently asked to select one picture from six to eight possible choices that would accurately complete the visual matrix. A participant’s score was the total number of correct answers. Cronbach’s α in our sample was 0.78.

#### 2.2.2. Reading

Chinese character recognition. The Chinese Character Recognition Measure and Assessment Scale for Primary School Children [36] is a commonly used task to screen for reading difficulties among Mandarin-speaking Chinese children [39]. In this test, children had to recognize 194 characters using each character in a phrase or word. The score of each character was weighted by its difficulty coefficient. The sum of the scores of characters used correctly was the score in this test. Cronbach’s α in our sample was 0.98.

Chinese character identification. The Chinese character identification task consists of three recognition cards printed with a total of 156 Chinese characters, which are arranged in terms of increasing difficulty [40]. The test was discontinued after 10 consecutive errors, and a participant’s score was the total number of Chinese characters read correctly. Cronbach’s α in our sample was 0.91.

#### 2.2.3. Mathematics

Math computation. The math computation subtest of the Wide Range Achievement Test-4 (WRAT-IV) [37] is a commonly used test to screen for mathematical difficulties. The test required children to complete 80 calculation questions with increasing difficulty within 30 min. The final score is the total number of correct responses. Cronbach’s α in our sample was 0.85.

Math problem solving. The arithmetic subtest in the Chinese version of the Wechsler Intelligence Scale for Children–Fourth Edition [41] was used to test children’s math problem-solving skills. Children were asked to solve 31 mathematical word problems of increasing difficulty presented on paper. A trained experimenter read each word problem to children to prevent comprehension problems. A participant’s score was the total number they got correct. Cronbach’s α in our sample was 0.52.

#### 2.2.4. Working Memory

##### Phonological Loop

Word span forward. In this task, children were asked to repeat strings of nonsense Chinese characters/syllables presented by an audio player at a rate of one character/syllable per second, following the same order they had heard them. The length of the character/syllable strings ranged from 2 to 11 characters/syllables. The final score was the number of items answered correctly (max = 30). Cronbach’s α in our sample was 0.77.

Digit span forward. This test is similar to the word span task mentioned above, except that strings of digits were used instead of nonsense Chinese characters/syllables. The strings of digits came from the Chinese version of the Wechsler Intelligence Scale for Children–Fourth Edition [41]. Children were required to repeat the digit strings presented on an audio player at a rate of one digit per second, following the same order they had heard them. The length of digit strings ranged from 2 to 11. The final score was the number of items answered correctly (max = 20). Cronbach’s α in our sample was 0.80.

##### Central Executive

Complex word span. This task was adapted from the complex reading span paradigm of Daneman and Carpenter [42]. Children were asked to first read the sentences presented on the screen aloud and immediately judge whether the meaning of the sentence was correct, and then remember the last word in that sentence at the same time. At the end of a set of sentences, children were told to recall the last word in the sentences in the same order they were presented. The task began with two sentences and gradually increased to eight sentences. If a child made three consecutive errors at a certain level, the task stopped. The final score was the number of items answered correctly (max = 21). Cronbach’s α for the current sample was 0.79.

Complex digit span. This task was adapted from the complex computation span task developed by Salthouse and Meinz [43]. In each trial, children were asked to read aloud simple operations (e.g., 2 + 3 = ?) presented on the screen and provided their answers. After the children provided their answer, a single-digit number was presented on the screen for 1 s, and the children were required to remember this number. After completing a set of arithmetic operations, the children were asked to recall the remembered numbers in the order in which they were presented. The task started with two operations and gradually increased to ten operations. If a child made three consecutive errors at a certain level, the task stopped. The final score was the number of items answered correctly (max = 27). Cronbach’s α for the current sample was 0.89.

##### Updating

Word updating. This task was adapted from the 2-back task [44]. In this task, a sequence of two-syllable words is presented on the screen, with each word displayed for 2 s. Children were asked to judge whether the current word and the word before the previous word belonged to the same category (e.g., animals). If it was from the same category, children were asked to press z on the keyboard; if not, they pressed n on the keyboard. The correct rate of children’s responses was used as an indicator of their updating ability.

Digit updating. This task was also adapted from the 2-back task [44]. In this task, a sequence of single digits was presented on the screen, with each digit displayed for 2 s. Children were asked to judge whether the current digit and the digit before the previous digit were the same. If it was the same, children were asked to press z; if not, the n button was pressed on the keyboard. The correct rate of children’s responses was used as an indicator of their updating ability.

#### 2.2.5. Mother’s Education

We asked mothers to report on their highest achieved education by selecting one of six options: 1 = primary school, 2 = junior middle school, 3 = senior middle school, 4 = three-year college, 5 = four-year university, and 6 = graduate school.

## 3. Results

Before conducting the analyses, we examined whether the data met the assumptions of the analysis of covariance (conditional variance homogeneity, conditional normality, regression slope homogeneity, linear relationship, etc.; see Huitema [45]). Variables that did not meet the assumptions were transformed using the Box-Cox transformation with the MedCalc 22.014 software package (MedCalc Software, Ostend, Belgium). Even after this transformation, the assumption of homogeneity of variance was not met in word span forward tasks (*F* = 3.921, *p* = 0.011). Because ANCOVA is robust to violations of either normality or homoscedasticity [46], we chose to perform the group comparisons with the conventional ANCOVA, and for word span forward tasks, we also supplemented the results with the Kruskal–Wallis test, which is recommended when there is a violation of normality.

A series of two (RD vs. non-RD) × 2 (MD vs. non-MD) analyses of covariance (ANCOVA) were conducted to examine group differences across working memory tasks, with gender and non-verbal IQ as covariates. The use of 2 × 2 ANCOVA, rather than one-way ANCOVA with four groups, was considered necessary to test whether groups with learning disabilities exhibited domain-specific deficits or domain-general deficits in working memory and to explore whether the performance of children with comorbid RDMD showed the sum of single difficulty deficits (additive deficits), or exhibited distinct patterns of deficits (over-additive deficits). Post hoc pairwise comparisons were performed after the examination of the main effects and interactions. Means and standard deviations for each working memory task across groups, alongside the results of the ANCOVAs, are shown in Table 2.

### 3.1. Phonological Loop

For both the word span forward and number span forward tasks, the results show that the effects of reading difficulties were significant (word span forward task: *F* (1, 92) = 30.505, *p* < 0.001, η_p_^2^ = 0.249; digit span forward task: *F* (1, 92) = 19.970, *p* < 0.001, η_p_^2^ = 0.178). No significant effects of mathematics difficulties and no significant interaction of RD × MD was found (η_p_^2^s ≤ 0.063), suggesting that the comorbid RDMD group had an additive combination of single deficits. The result of the Kruskal–Wallis test also revealed a significant effect of reading difficulties (*H* = 22.821, *p* < 0.001), whereas the effect of mathematical difficulties was not significant (*H* = 1.476, *p* = 0.224). Simple contrasts of word span indicated that the groups with reading difficulties (RD only and comorbid RDMD) performed significantly worse than the groups without reading difficulties (controls and MD only) (*p*s < 0.05). For digit span forward tasks, all groups with reading difficulties (RD only and comorbid RDMD) performed significantly worse than the control group (*p*s < 0.05). These results suggest that the phonological loop is particularly related to reading difficulties.

### 3.2. Central Executive

For the complex word span task, the effects of reading difficulties (*F* (1, 92) = 26.212, *p* < 0.001, η_p_^2^ = 0.222) and mathematics difficulties (*F* (1, 92) = 8.571, *p* < 0.05, η_p_^2^ = 0.085) were both significant, whereas the interaction was not (η_p_^2^ = 0.006), indicating that the comorbid RDMD group showed additive deficits of single difficulties. Simple contrasts showed that groups with reading difficulties (RD only and comorbid RDMD) performed significantly worse than the control group (*p*s < 0.001), and the comorbid RDMD group performed significantly worse than the MD-only group (*p* < 0.05). To further investigate whether the complex word span task is related to domain-specific difficulties, we used the complex digit span task as a covariate. The main effect of reading difficulties persisted (*F* (1, 91) = 19.395, *p* < 0.001, η_p_^2^ = 0.176), whereas the effect of mathematics difficulties disappeared (η_p_^2^ = 0.007), supporting the concept that reading difficulties showed domain-specific deficits in complex word span tasks.

For the complex digit span task, only mathematics difficulties showed a significant main effect (*F* (1, 92) = 28.172, *p* < 0.001, η_p_^2^ = 0.234), with no significant main effect for reading difficulties, and no significant RD × MD interaction (η_p_^2^s ≤ 0.057), suggesting that the comorbid RDMD group had an additive combination of single deficits. Simple contrasts revealed that the groups with mathematics difficulties (MD only and comorbid RDMD) performed significantly worse than the control group (*p*s < 0.001), and the comorbid RDMD group performed significantly worse than the RD-only group (*p* < 0.05) supporting the concept that mathematics difficulties show domain-specific deficits in complex digit span tasks.

### 3.3. Updating

For the word updating task, only the main effect of reading difficulties was significant (*F* (1, 92) = 9.226, *p* < 0.05, η_p_^2^ = 0.091). No significant main effect for mathematics difficulties and no significant interaction of RD × MD was found (η_p_^2^s ≤ 0.054), indicating that the comorbid RDMD group exhibited an additive combination of single deficits. Simple contrasts indicated that the comorbid RDMD group performed significantly worse than the groups without reading difficulties (controls and MD only) (*p*s < 0.05), which suggests that reading difficulties are associated with domain-specific deficits in word updating tasks.

For the digit updating task, only the main effect of mathematics difficulties was significant (*F* (1, 92) = 15. 252, *p* < 0.01, η_p_^2^ = 0.142), with no significant main effect of reading difficulties, and no significant RD × MD interaction (η_p_^2^s ≤ 0.062). Simple contrasts indicated that the comorbid RDMD group performed significantly worse than the groups without mathematics difficulties (controls and RD only) (*p*s < 0.01), which suggests that mathematics difficulties are associated with domain-specific deficits in digit updating tasks.

## 4. Discussion

The purpose of this study was to examine what component(s) of working memory may be impaired in children with comorbid difficulties in reading and mathematics. After matching the groups based on relevant reading and mathematics tasks, we compared the performance of children with RD, MD, and comorbid RDMD to that of chronological-age controls to measure phonological loop, central executive, and updating tasks. The results indicate that children with reading difficulties (RD only and comorbid RDMD) exhibit deficits in both word span and digit span forward tasks within the phonological loop compared to children without reading difficulties (MD only and controls). This suggests a closer connection between the phonological loop task and reading difficulties (a finding that has already been reported in the literature; see [47,48]). In terms of central executive and updating tasks, children with learning difficulties exhibited domain-specific deficits. Specifically, children with reading difficulties (RD only and comorbid RDMD) performed worse in word-related tasks compared to those without reading difficulties (MD only and controls), and children with mathematical difficulties (MD only and comorbid RDMD) performed worse in number-related tasks compared to those without mathematical difficulties (RD only and controls). In children with comorbid RDMD, deficits in the phonological loop, central executive, and updating tasks appeared to be an additive combination of deficits in the RD and MD groups, thus supporting the correlated liability model of comorbidity. Taken together, our findings suggest that the type of working memory task researchers use when searching for underlying working memory deficits in individuals with reading or mathematics difficulties may determine whether significant group differences are found.

In Baddeley’s working memory model, the phonological loop is dedicated to the temporary holding of verbal information, thus making it more reliant on phonological processing than on the central executive component [7,49]. One of the core deficits in reading difficulties is in phonological processing [47], making its strong association with the phonological loop understandable. For children with mathematics difficulties, although earlier studies have identified deficits in the phonological loop [26,50], this could be attributed to the inadequate matching of reading abilities between children with mathematics difficulties and controls. A meta-analysis revealed that in studies where reading ability was matched, the difference in phonological loop between MD and the controls was 0.48 *SD* smaller compared to studies without reading being matched [51]. In studies that matched their groups with reading, children with mathematics difficulties did not exhibit significant deficits in the phonological loop (e.g., [20,52]).

Our findings regarding the central executive task are consistent with the findings of the meta-analysis by Peng et al. [12]. Children with reading difficulties and those with mathematical difficulties both displayed deficits in complex word span tasks, while in terms of the complex digit span task, children with mathematical difficulties exhibited more severe deficits than children with reading difficulties. Following the additional control for the complex digit span task, our study found that only the RD groups exhibited deficits in the complex word span task, thereby providing support for domain-specific difficulties in the central executive task. In Baddeley’s working memory model, the central executive not only regulated the phonological loop but also directed attention to relevant information, activated representations in long-term memory, and inhibited irrelevant information and inappropriate actions [13]. The theory of long-term working memory suggests that domain-specific expertise greatly influences working memory’s capacity within domains [53], particularly when cognitive load is heightened [54]. Compared to the phonological loop, lacking domain-specific knowledge is more likely to affect central executive tasks in the group with learning difficulties. Similarly, updating requires monitoring, encoding task-relevant information, and appropriately revising items stored in the working memory [14]. In a factor analysis encompassing all working memory and executive functions, updating and working memory were loaded onto the same factor [55], closely associated with reading ability and mathematics performance. Thus, it is reasonable to expect that updating and central executive tasks together are related to domain-specific difficulties.

Similar to other studies, our findings also support the correlated liability model, according to which the comorbid RDMD group displays an additive combination of deficits in working memory in the RD and MD groups [25,26,27,33,34]. This perspective is also corroborated by the findings of neuroimaging studies, as no distinct neural activation patterns specific to RDMD have been identified [56], at least not in tasks related to phonological working memory [16]. In the ICD-10, a mixed disorder of scholastic skills is categorized as a subtype within specific reading disorders, and this relevant classification still requires to be discussed in terms of etiology [57].

Some limitations of the present study should be mentioned. First, its cross-sectional nature did not allow us to draw any causal inferences. A longitudinal study could offer more insights into the development of working memory deficits in both single and comorbid difficulty groups. Second, we did not administer measures of the visuospatial sketchpad in our study. Unfortunately, we were only given a specific amount of time to test our participants, and we had to make a tough decision as to what components to measure. A future study should also include measures of the visuospatial sketchpad. Third, our sample consisted of fifth graders, and our results may not generalize to other grade levels. Finally, according to Gelman et al. [58], in order to estimate the interaction with the same size as the main effect, a sample size four times larger is required. Given that we only had 26 participants in the comorbid RDMD group, we might not have enough power to detect a significant interaction, should there be one. Future studies should repeat our analyses with a much larger sample size.

Despite these limitations, our findings have implications for testing and teaching. In particular, the present results provide us with more insights into the selection of working memory tasks when searching for underlying working memory deficits in children with reading and mathematics difficulties. Furthermore, accurately identifying the underlying deficits of students with learning difficulties can help teachers develop more customized instructional strategies for each group of children.

## 5. Conclusions

We found that the phonological loop is particularly related to reading difficulties, while the central executive and updating tasks are more closely related to domain-specific difficulties. Moreover, working memory deficits in the comorbid RDMD group showed an additive combination of single difficulties in both RD and MD groups. Taken together, these findings suggest that different components of working memory may impact reading and mathematics difficulties differently. In conclusion, the findings of this study contribute to our understanding of the nature of working memory deficits in children with reading and mathematics difficulties, particularly for the comorbid RDMD group, which demonstrates an additive combination of the deficits in working memory experienced by children with either reading or mathematics difficulties.

## Figures and Tables

**Table 1:** Sample characteristics.

Variables	Control (*n* = 27)		RD (*n* = 21)		MD (*n* = 24)		Comorbid RDMD (*n* = 26)		*F* (3, 94)	*p*	η_p_^2^	Post Hoc (Bonferroni)
	Mean	*SD*	Mean	*SD*	Mean	*SD*	Mean	*SD*				
Age (months)	131.52	4.13	131.48	3.40	132.50	6.41	130.81	4.23	0.547	0.651	0.017	RDMD = RD = MD = TD
Non-verbal IQ	43.19	4.77	43.19	5.51	41.67	6.21	41.92	4.74	0.567	0.638	0.018	RDMD = RD = MD = TD
Gender (female percentage)	66.7%		33.3%		25%		23.1%					RDMD = MD < TD RDMD = MD = RD RD = TD
Mother’s education level (senior middle school level and above)	40.7%		33.3%		41.7%		38.5%					RDMD = RD = MD = TD
Chinese character recognition	1941.68	96.09	1312.33	138.96	1855.54	109.60	1232.74	115.85	252.157	<0.001	0.889	RDMD = RD < MD = TD
Chinese character identification	126.82	7.23	109.95	7.61	122.63	4.63	107.85	11.21	33.513	<0.001	0.517	RDMD = RD < MD = TD
Math computation	50.85	4.22	48.10	1.84	39.58	2.36	37.31	4.75	83.334	<0.001	0.727	RDMD = MD < RD = TD
Math problem solving	24.96	1.19	23.57	1.54	21.92	1.64	21.50	2.64	19.318	<0.001	0.381	RDMD = MD < RD = TD

Note. Control, chronological-age controls; RD, reading difficulties; MD, mathematics difficulties; comorbid RDMD, comorbid reading and mathematics difficulties.

**Table 2:** Means, standard deviations, and 2 × 2 ANCOVAs for working memory tasks.

Working Memory Tasks	Control (*n* = 27)		RD (*n* = 21)		MD (*n* = 24)		Comorbid RDMD (*n* = 26)		Main Effect of RD		Main Effect of MD		RD × MD Interaction		RD Deficit (vs. Control)	MD Deficit (vs. Control)	RD&MD Deficit (vs. Control)
	Mean	*SD*	Mean	*SD*	Mean	*SD*	Mean	*SD*	*F*	η_p_^2^	*F*	η_p_^2^	*F*	η_p_^2^	Cohen’s *d* (95% CI)	Cohen’s *d* (95% CI)	Cohen’s *d* (95% CI)
Word span forward	12.37	2.91	9.52	1.47	11.38	2.06	9.15	2.29	30.505 ***	0.249	1.417	0.015	0.063	0.001	1.194 (0.350, 2.038)	0.306 (−0.499, 1.110)	1.396 (0.559, 2.233)
Digit span forward	12.56	2.71	10.10	2.66	10.54	1.93	9.15	1.52	19.970 ***	0.178	6.234	0.063	0.738	0.008	1.101 (0.262, 1.940)	0.709 (−0.105, 1.523)	1.456 (0.615, 2.298)
Complex word span	7.00	3.09	4.24	2.00	4.75	1.80	3.42	1.55	26.212 ***	0.222	8.571 *	0.085	0.575	0.006	1.215 (0.370, 2.060)	0.780 (−0.037, 1.597)	1.683 (0.825, 2.541)
Complex digit span	11.04	4.28	8.10	4.88	5.71	4.49	4.92	3.05	5.543	0.057	28.172 ***	0.234	1.748	0.019	0.759 (−0.065, 1.583)	1.403 (0.554, 2.253)	1.618 (0.765, 2.471)
Word updating	0.79	0.09	0.76	0.09	0.77	0.11	0.69	0.13	9.226 *	0.091	5.267	0.054	1.577	0.017	0.369 (−0.444, 1.183)	0.230 (−0.573, 1.034)	1.117 (0.297, 1.938)
Digit updating	0.81	0.09	0.79	0.11	0.75	0.11	0.67	0.14	6.107	0.062	15.252 **	0.142	0.632	0.007	0.347 (−0.466, 1.160)	0.668 (−0.145, 1.481)	1.343 (0.509, 2.177)

Note. Control, chronological-age controls; RD, reading difficulties; MD, mathematics difficulties; comorbid RDMD, comorbid reading and mathematics difficulties; covarying for gender and non-verbal IQ; *p*-values have been corrected using the Bonferroni correction: * *p* < 0.05; ** *p* < 0.01; *** *p* < 0.001.

## Data Availability

The datasets generated or analyzed during the current study are available from the corresponding author upon reasonable request.
